# Delivery of a mitochondria‐targeted antioxidant from biocompatible, polymeric nanofibrous scaffolds

**DOI:** 10.1002/2211-5463.13032

**Published:** 2020-12-08

**Authors:** Yasaman Hamedani, Rayane Brinck Teixeira, Catherine Karbasiafshar, Peter Wipf, Sankha Bhowmick, M. Ruhul Abid

**Affiliations:** ^1^ Department of Mechanical Engineering University of Massachusetts Dartmouth North Dartmouth MA USA; ^2^ Division of Cardiothoracic Surgery Department of Surgery Cardiovascular Research Center Rhode Island Hospital Warren Alpert Medical School of Brown University Providence RI USA; ^3^ Department of Chemistry University of Pittsburgh PA USA; ^4^ Department of Pharmaceutical Sciences University of Pittsburgh PA USA; ^5^ Department of Bioengineering University of Pittsburgh PA USA

**Keywords:** biopolymer, drug delivery, electrospinning, endothelial cells, mitochondrial antioxidants, nanofibrous scaffold

## Abstract

Cardiovascular disease has been associated with increased levels of reactive oxygen species (ROS). Recently, we have shown that a critical balance between cytosolic ROS and mitochondrial ROS is crucial in cardiovascular health and that modulation of mitochondrial ROS helps prevent detrimental effects of cytosolic ROS on endothelial cells (EC) in transgenic animals. Here, we report the development of a controlled delivery system for a mitochondria‐targeted antioxidant, JP4‐039, from an electrospun scaffold made of FDA‐approved biocompatible polymeric nanofibers. We demonstrate that the active antioxidant moiety was preserved in released JP4‐039 for over 72 h using electron paramagnetic resonance. We also show that both the initial burst release of the drug within the first 20 min and the ensuing slow and sustained release that occurred over the next 24 h improved tube formation in human coronary artery ECs (HCAEC) *in vitro*. Taken together, these findings suggest that electrospinning methods can be used to upload mitochondrial antioxidant (JP4‐039) onto a biocompatible nanofibrous PLGA scaffold, and the uploaded drug (JP4‐039) retains nitroxide antioxidant properties upon release from the scaffold, which in turn can reduce mitochondrial ROS and improve EC function *in vitro*.

## Graphical abstract

Using electrospinning, we fabricated a biocompatible nanofibrous scaffold with mitochondrial antioxidant JP4‐039. Electron paramagnetic resonance spectroscopy tracked an initial burst release of JP4‐039 within the first 20 min followed by a sustained release over 72 h. The released drug reduced mitochondrial reactive oxygen species and induced migration and tube formation in human coronary artery endothelial cells, suggesting therapeutic potential of the implants.

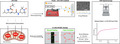

**Abbreviations**
EC: endothelial cell
EPR: electron paramagnetic resonance
FTIR: Fourier transform infrared spectroscopy
JP4‐039: 4‐[[(3*E*,5*S*)‐5‐[[(1,1‐dimethylethoxy)carbonyl]amino]‐7‐methyl‐1‐oxo‐3‐octen‐1‐yl]amino]‐2,2,6,6‐tetramethyl‐1‐piperidinyloxy
MI: myocardial infarction
PLGA: polylactide‐co‐glycolide acid
ROS: reactive oxygen species
SEM: scanning electron microscopy
TFE: 2,2,2‐trifluoroethanol

Cardiovascular disease, including ischemic heart disease and myocardial infarction (MI), is the leading cause of death in the world. One of the major consequences of MI is death of cardiomyocytes leading to cardiac remodeling, fibrosis, and heart failure [1]. There are major efforts undergoing to improve blood supply to the ischemic myocardium by inducing the growth or sprouting of new blood vessels, that is, angiogenesis, from the existing coronary vessels. Recent work from our laboratory and others showed that reactive oxygen species (ROS) plays a major role in determining coronary vascular endothelial cell (EC) proliferation, migration, and tube formation *in vitro* [2, 3, 4, 5]. ECs have several intracellular sources for ROS, including membrane‐bound NADPH oxidases, mitochondria, peroxisome, and cytochrome P450 [6, 7, 8, 9, 10, 11, 12]. Whereas the increase in ROS is believed to be harmful for the cardiovascular system, the use of global antioxidants to eliminate excessive ROS has resulted in increased mortality [13]. A recent report from our laboratory demonstrated that the detrimental effects of a chronic increase in ROS can be abrogated by specific reduction in the levels of mitochondrial ROS in ECs [14]. Although ECs are dependent on glycolysis, not oxidative phosphorylation, for its ATP synthesis, mitochondria play an important role in fatty acid oxidation‐dependent nucleotide synthesis that is required for EC proliferation [15]. Mitochondrial ROS balance is critical for maintenance of membrane potential, which is crucial for mitochondrial function. Thus, regulating mitochondrial ROS in EC insures the optimal function of mitochondria. Taken together, it appears that targeted delivery of antioxidants to mitochondria may help maintain EC health during oxidative stress. Achieving appropriate EC health by modulating mitochondrial ROS would insure coronary angiogenesis in ischemic myocardium and thus would help recover cardiac function in myocardial ischemia or the post‐MI heart. In recent years, there has been a great interest in developing antioxidants which are capable of targeting mitochondria and providing their radical scavenging activity inside the matrix to reduce mitochondrial ROS [16, 17, 18, 19]. JP4‐039 is one such derivative with a delivery core based on the antibiotic gramicidin S, targeting the lipid bilayer of the mitochondrial membrane. JP4‐039 enters the matrix and provides radical and electron scavenging activity by virtue of its nitroxide radical containing 4‐amino‐TEMPO payload [20, 21]. Moreover, JP4‐039 has demonstrated efficacy as a radiation protector and mitigator in quiescent cells [22], in a mouse model of radiation‐induced skin damage by reducing ROS in mitochondria [23], and as radical protector for colony‐forming unit‐granulocyte‐erythroid‐megakaryocyte‐monocytes [24].

Recent studies have shown the critical requirement of basal ROS levels in ECs to provide cardioprotection as well as differentiation and excitation‐contraction coupling for cardiomyocytes [1, 14]. Our previous study using ECs demonstrated that a critical timed reduction in mitochondrial ROS improved endothelial function and proliferation during oxidative stress [14]. Although the exact dosing of the mitochondrial antioxidant drug required *in vivo* has yet to be established, it is clear that the antioxidant delivery requires a controlled release as opposed to a regular injectable dosage. Therefore, designing a robust and flexible drug delivery system is of paramount importance to maintain an optimal balance between subcellular ROS.

Electrospinning is a versatile nanofabrication technique in which solid fibers can be obtained from polymeric solutions. This technique has been successfully used as a controlled drug delivery system, being able to provide a suitable amount of a desired drug to the target area [25, 26, 27, 28]. The enclosed drug can be released from the nanofibrous scaffolds by desorption from the surface, diffusion into the surrounding media or by degradation of the nanofibrous scaffold. Control over the release of the drug can be obtained by manipulating the morphology of the nanofibers at the nanoscale level, namely, fiber diameter, density of nanopores, and alignment of the fibers, by choosing the proper polymer to electrospin, or by providing externally manipulatable resources in the fibers to be able to externally control the release [29, 30, 31, 32, 33].

In this study, we examined whether electrospinning method to upload JP4‐039 onto an FDA‐approved biocompatible polymer PLGA‐based nanofibrous scaffold will retain the antioxidant property of JP4‐039 when released from the scaffold over a period of time. As a first step, we have successfully encapsulated the mitochondria‐targeting antioxidant JP4‐039 into PLGA 85 : 15 electrospun fibers. We then evaluated the release pattern of the drug, JP4‐039, from the scaffold, the presence and activity of the radical scavenging moiety in the released drug using electron paramagnetic resonance (EPR). We also examined the effects of the released mitochondrial antioxidant activity JP4‐039 on tube formation in human coronary artery EC (HCAEC) *in vitro*.

## Materials and methods

### Polymers and reagents

Polylactide‐co‐glycolide acid (PLGA) Resomer LG 857 (Boehringer Ingelheim Pharma GmbH & Co.KG Company, Ingelheim, Germany). 2,2,2‐Trifluoroethanol (TFE) > 99% (Sigma‐Aldrich Company, St. Louis, MO, USA). JP4‐039 has been provided by P. Wipf's laboratory in Pittsburgh University and was prepared as previously reported [33]. Human Coronary Artery Endothelial Cells (HCAEC) (ATCC, Manassas, VA, USA). EGM‐2MV media (Lonza, Morristown, NJ, USA), DPBS (Thermo Fisher Scientific, Waltham, MA, USA). Cultrex^®^ basement membrane extract (BME; R&D Systems, Minneapolis, MN, USA). 16‐well chamber slide (Thermo Fisher Scientific).

### Electrospinning

A lab‐built electrospinning device was used consisting of a high‐voltage power supply (Glassman Co, EH‐Series, High Bridge, NJ, USA), syringe pump (Braintree Scientific Co, BS‐8000, Braintree, MA, USA), and a flat aluminum plate covered with a thin aluminum foil. All the different parts were enclosed in an environmental‐controlled chamber capable of providing various relative humidity range (16% up to 90%). PLGA with a lactic acid : glycolic acid ratio of 85 : 15 was dissolved in trifluoroethanol (TFE) solvent in 2 wt% concentration to achieve a proper spinnable concentration. Various electrospinning parameters were utilized and adjusted to reach the optimized electrospinning conditions, by which continuous bead‐free fibers can be obtained. The solutions were placed inside of a 1‐mL glass syringe (Hamilton, Reno, NV, USA) connected to a flat tip 22‐gauge metal needle. The syringe was placed inside of the flow pump, 17 cm away from the stationary aluminum deposition collector, which was covered by a thin aluminum foil. The flow rate of the solution was set at 0.2 mL·h^−1^ and the applied voltage to the tip of the needle was 15 kV. The collector was completely grounded. The deposition time was monitored accurately to have samples with the exact thicknesses.

JP4‐039 was dissolved in a PLGA 85 : 15‐TFE solution in 2% concentration and the obtained solution was electrospun via a physical blending method and the fibers got deposited on the aluminum foil by monitoring the deposition time as well as the weight of the deposited scaffold, to reach to the proper thickness.

### Scanning electron microscopy

The morphology of the electrospun samples was monitored using Hitachi SU5000 scanning electron microscopy (Hitachi, Clarksburg, MD, USA) at different accelerating voltages of 5–20 kV. Briefly the samples were placed on the double‐sided carbon tape, mounted on the Hitachi scanning electron microscopy stubs, and monitored. The average fiber diameters were measured using imagej software (NIH, Bethesda, MD, USA).

### Fourier transform infrared spectroscopy

Fourier transform infrared (FTIR) spectroscopy was utilized to confirm the perseverance of the chemical structure of the polymers and the drug after being exposed to the electrostatic field during electrospinning procedure. The fibrous scaffold, with and without the loaded JP4‐039, was ground finely and mixed with IR‐grade potassium bromide (KBr) in ratio of 5 : 95. The ground mixture powder was then pressed into a pellet for spectral measurements, scanned for 500 times with the resolution of 2 cm^−1^ against blank KBr pellet as the reference. The FTIR spectra were obtained using Digilab Excalibur FTS3000MX and the Win‐IR Pro Program (Digilab, Hopkinton, MA, USA).

### Evaluation of radical scavenging active moiety of the encapsulated JP4‐039

In order to evaluate the perseverance of the radical scavenging activity of JP4‐039 after being exposed to an electrostatic field via electrospinning, Bruker BioSpin's e‐scan electron paramagnetic resonance spectroscopy (Bruker, Billerica, MA, USA) was used to detect the triplet nitroxide peaks of the dissolved JP4‐039‐loaded scaffold in TFE. For this purpose, a 50 μL aliquot of the TFE solution in which the JP4‐039‐loaded scaffold was dissolved was placed inside of micropipettes. The micropipette was placed in the quartz EPR tube. The aliquot was scanned 100 times, under the following conditions; 3487 G center field, 0.54 G modulation amplitude, 38.5 mW microwave power, and 2.62 s sweep time. The triplet nitroxide radical peaks were obtained using winepr software and plotted using Microsoft Excel [34].

### Calibration curve of JP4‐039

To be able to quantify the amount of the released JP4‐039 from the scaffolds, a calibration curve of the drug was obtained using EPR spectroscopy. Three different series of 11 known concentrations of JP4‐039 (0.1, 0.5, 1, 2.5, 5, 7.5, 10, 12.5, 15, 17.5, and 20 μm) were prepared in TFE and analyzed using Bruker BioSpin's E‐scan EPR spectroscopy. Briefly, a 50‐μL aliquot of each solution was placed inside of a micropipette. The micropipette was placed in the quartz EPR tube. Each of the aliquots was scanned 100 times, under the following conditions; 3487 G center field, 0.54 G modulation amplitude, 38.5 mW microwave power, and 2.62 s sweep time. The triplet nitroxide radical peaks were obtained using winepr software. To get a quantitative measure of the drug, the area below the integral of the 1st derivative graph obtained by winepr software was measured using originpro software. Calibration curve of the drug was then plotted for drug concentration vs area below the peaks [35, 36].

### Drug release assay

The drug‐loaded fibrous electrospun mats were removed from the foil and used for drug release assay. For this purpose, scaffolds with the same dimensions and thicknesses (0.25 square cm) were placed in 200 μL PBS (pH 7.2) kept at 37 °C. In different time intervals; 5 min until 72 h, the release medium was collected completely and replaced with fresh PBS. 50 μL aliquots of each of the collected media was analyzed using Bruker BioSpin's e‐scan EPR spectroscopy and winepr software to detect the nitroxide triplet peaks of the released drug. The triplet peaks were quantified to determine the concentration of the released drug using the obtained calibration curve [35, 36].

### Measurement of mitochondrial ROS

Mito‐ROS were measured using MitoSOX Red assay as described in the manufacturer's protocol (Thermo Fisher Scientific). In short, JP4‐039 was first dissolved in pure trifluoroethanol (TFE) and then diluted in PBS to a stock solution of 250 µm JP4‐039 in 1% TFE in PBS. The stock solution was then diluted to a final concentration of 5 µm JP4‐039 in EGM2‐MV media. Another solution containing the same amount of vehicle only (no JP4‐039) was diluted in EGM2‐MV media and used as control. HCAECs were incubated with or without 5 μm of JP4‐039 for 1 h and 30 min and subject to Mito‐ROS measurement assay. Data were obtained by measurement of fluorescence in a microplate reader.

### Tube formation assay

In order to evaluate whether released JP4‐039 from the electrospun nanofibrous scaffolds was still capable of modulating HCAEC phenotype, *in vitro* tube formation assay as a function of EC migration and proliferation was performed. In brief, a stock solution containing the released JP4‐039 in PBS was diluted to a final concentration of 5 μm released JP4‐039 using EGM2‐MV media. The stock was obtained in two different experiments; first a 0.5 × 0.5 square centimeter scaffold was kept in 200 µL PBS for 24 h straight to be used as representative of the drug release which contained both initial burst release plus sustained release over a period of 24 h and named as ‘unwashed sample’. Secondly, a 0.5 × 0.5 square centimeter scaffold was placed in 200 µL PBS for 20 min; then, the medium containing the burst release drug was discarded, a fresh 200 µL PBS was added to the scaffold, and the medium containing released drug was collected after 24 h (prewashed sample). In parallel, PBS‐containing released vehicle from scaffolds without JP4‐039 was equally dissolved in the aforementioned media and used as control for both types of ‘pre‐washed’ and ‘unwashed’ samples. HCAECs were plated into a 16‐well chamber slide precoated with Cultrex^®^ basement membrane extract at a density of 1.7 × 10^4^ cells/well (passage 4). Cells were diluted with the EGM2‐MV media containing 5 μm released JP4‐039 for test wells or released vehicle for control wells prior to plating (total of 8 wells per group). Cells were kept in an incubator at 5% CO_2_ and 37 °C and imaged after 3 h using 4× magnification phase contrast in an inverted microscope (Eclipse Ts2; Nikon Instruments Inc., Melville, NY, USA). Number of tubes was measured using imagej software.

### Scratch assay

HCAEC were plated in a 48‐well plate at a density of 3.2 × 10^4^ cells/well (passage 5) and allowed to grow in an incubator at 5% CO_2_ and 37 °C until full confluency (48 h). Prior to starting the assay, 70 µm stock solutions released from scaffolds in PBS were diluted with EGM2‐MV media to a final concentration of 5 µm released JP4‐039 or an equal volume of released vehicle in 37 °C EGM2‐MV media. Then, a yellow (200 µL) tip was used to make a transversal scratch in the middle of each well. Cell debris was promptly washed with warm EGM2‐MV media and replaced by warm EGM2‐MV media containing either released vehicle or released JP4‐039 from scaffolds (total of 8 wells per group). Cells were kept in an incubator at 5% CO_2_ and 37 °C and imaged after 3 and 6 h using 4× magnification phase contrast in an inverted microscope (Eclipse Ts2; Nikon Instruments Inc.). The scratch area was measured in pixels using imagej software and expressed as the percent decrease in the scratch area relative to the total area [37].

### Statistical analysis

**Fig. 1:**
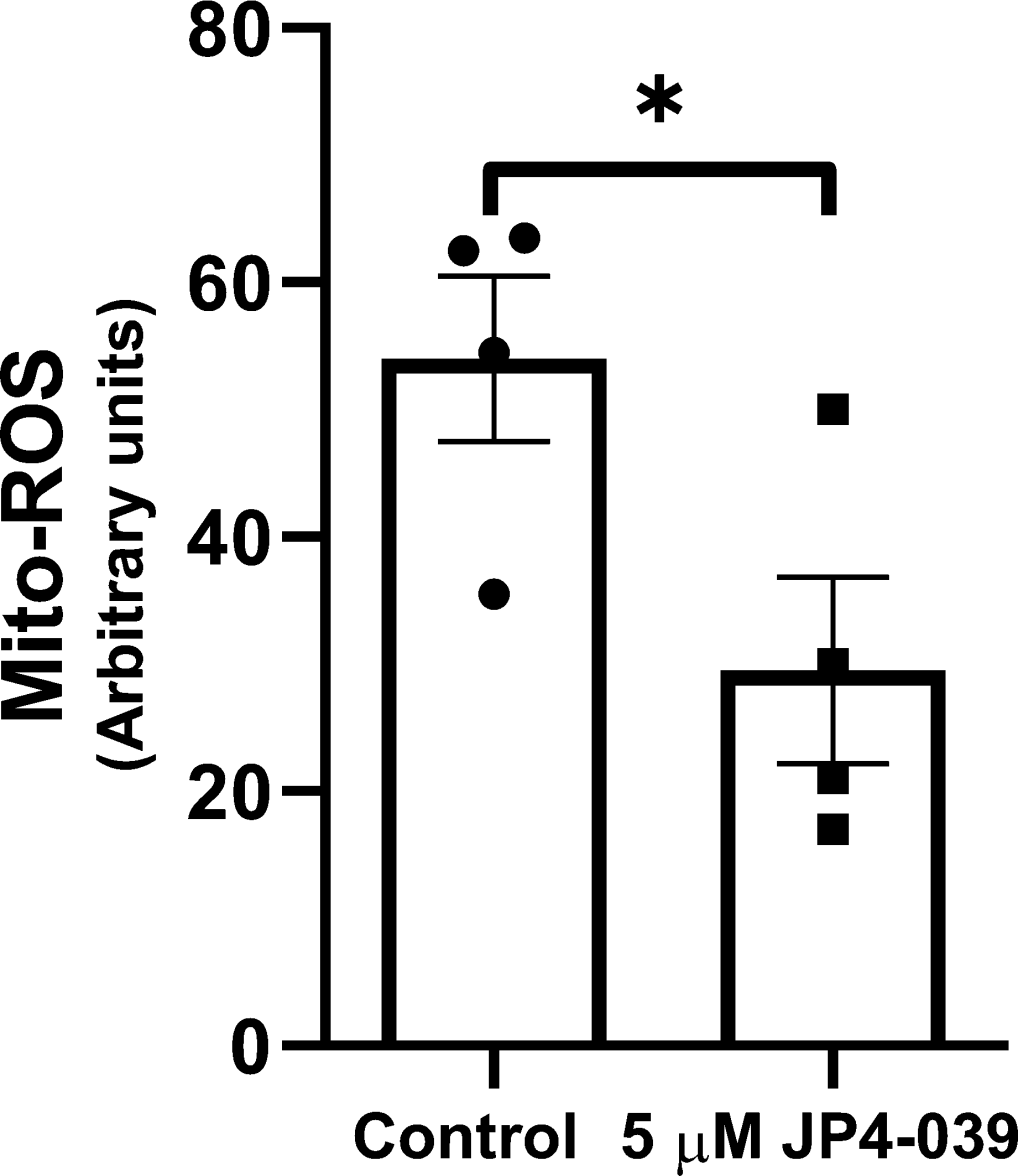
JP4‐039 reduces mitochondrial ROS in human coronary artery EC (HCAEC). HCAEC treated with 5 μm JP4‐039 significantly reduces mitochondrial ROS compared to vehicle control as measured by mitoSox assay. HCAECs were used between passages three to six. Data were analyzed using two‐tailed Student's *t*‐test considering a *P‐*value inferior to 0.05 as significant. Results are expressed as mean ± standard error of the mean. *n* = 4/group; *P < 0.05.

**Fig. 2:**
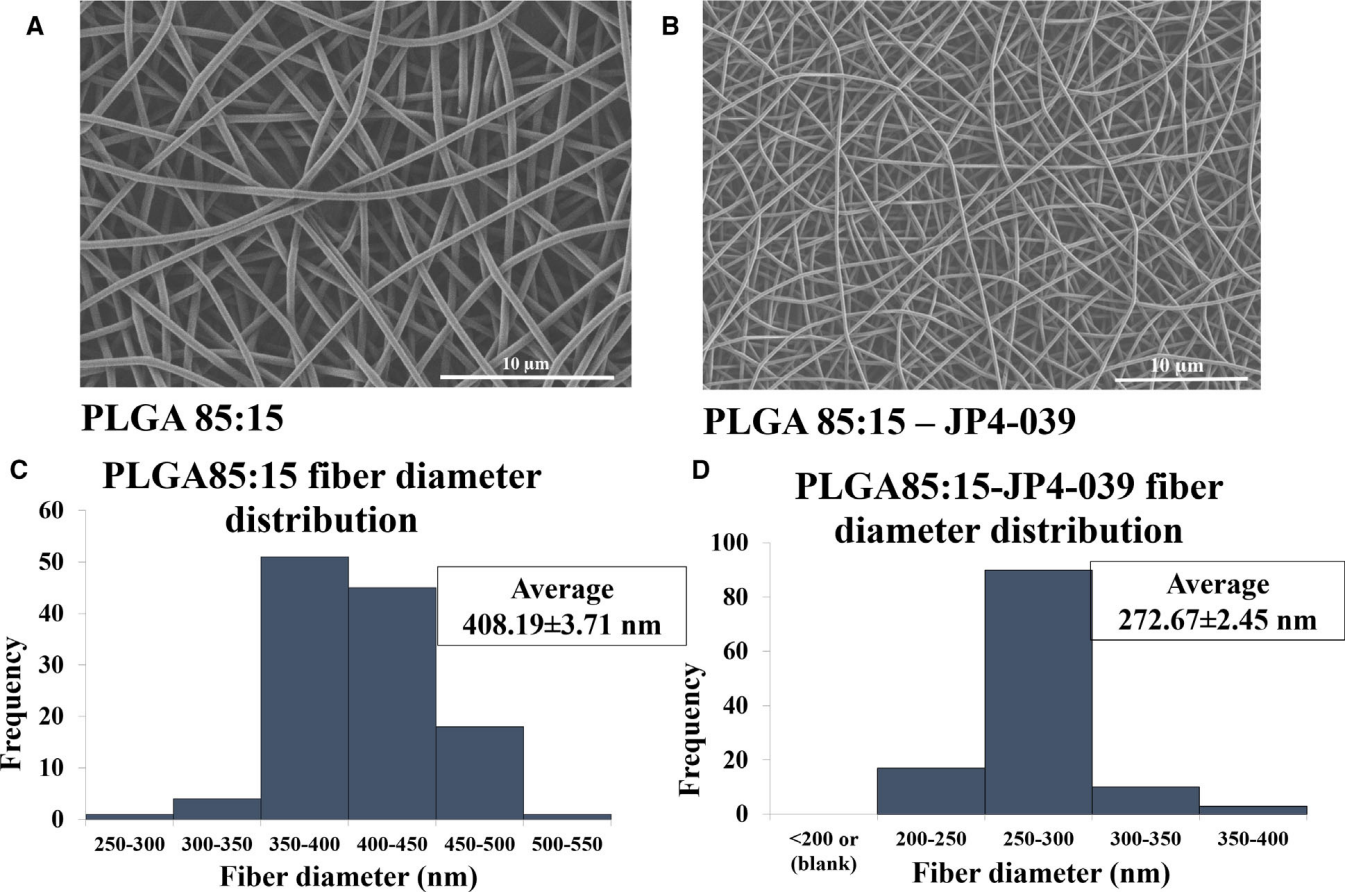
Uploading of mitochondrial antioxidant JP4‐039 onto PLGA polymer by electrospinning. Scanning electron microscopy pictures of (A) blank PLGA 85 : 15 scaffold, (B) JP4‐039‐loaded PLGA 85 : 15 scaffold showing continuous bead‐free morphology of the fabricated fibers. Diameter distribution of (C) blank PLGA 85 : 15 and (D) JP4‐039‐loaded PLGA 85 : 15 scaffolds demonstrating the uniform fibers with a normal diameter distribution, and reduction in the average fiber diameter by enclosure of the drug from 408.19 ± 3.71 nm to 272.67 ± 2.45 nm. Scale bar represents 10 µm.

**Fig. 3:**
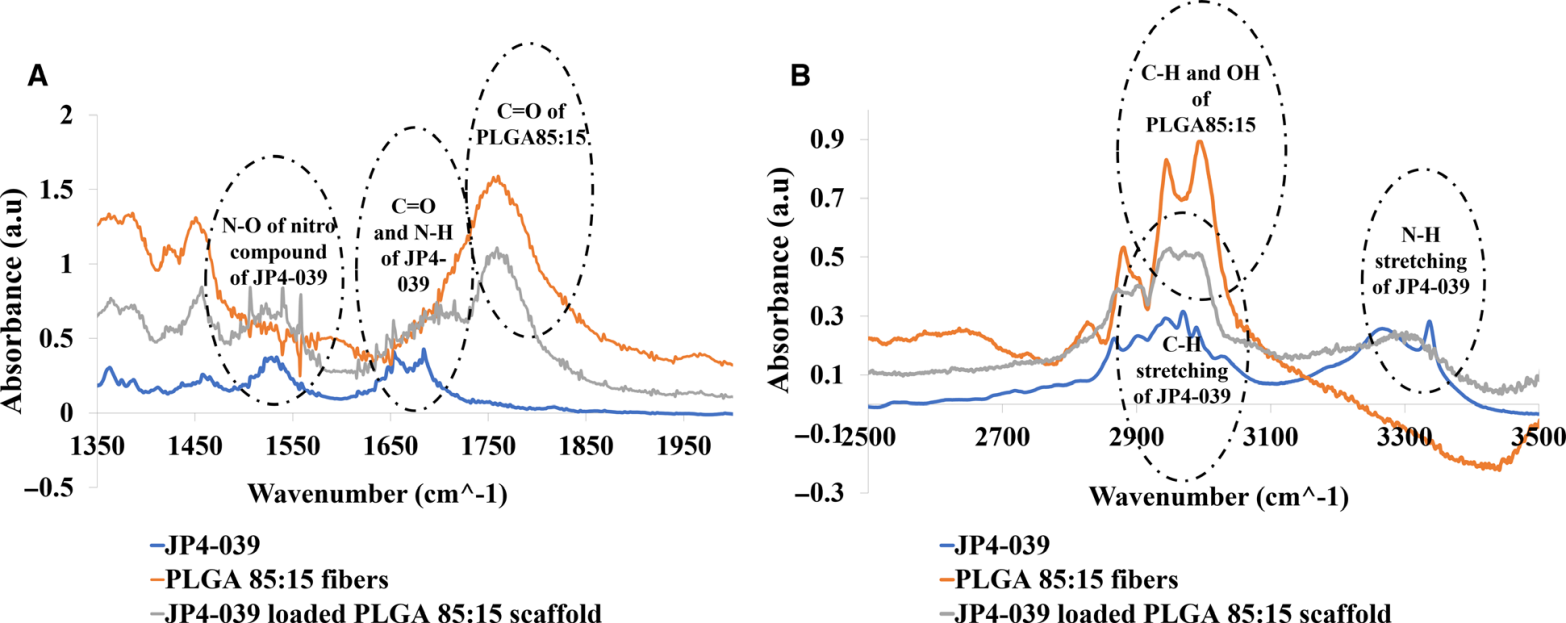
Fourier transform infrared spectra (FITR) of blank PLGA 85 : 15, JP4‐039 powder, and JP4‐039‐loaded PLGA 85 : 15 scaffold. Infrared spectra (A) from 1350 to 1950 cm^−1^, and (B) from 2500 to 3500 cm^−1^, demonstrating perseverance of the chemical structure of the drug postexposure to high electrostatic field during the fabrication technique by showing peaks at 3000, 2800, 1680, and 1650 cm^−1^ and at 1400–1500 cm^−1^, which correspond to C–H stretching in alkenes, C–H stretching at alkanes, C = O stretching and N–H bonds, N–O of the nitro compound of the drug, respectively, which are all observed in the JP4‐039 spectra and JP4‐039‐loaded PLGA85 : 15 fibers as well.

**Fig. 4:**
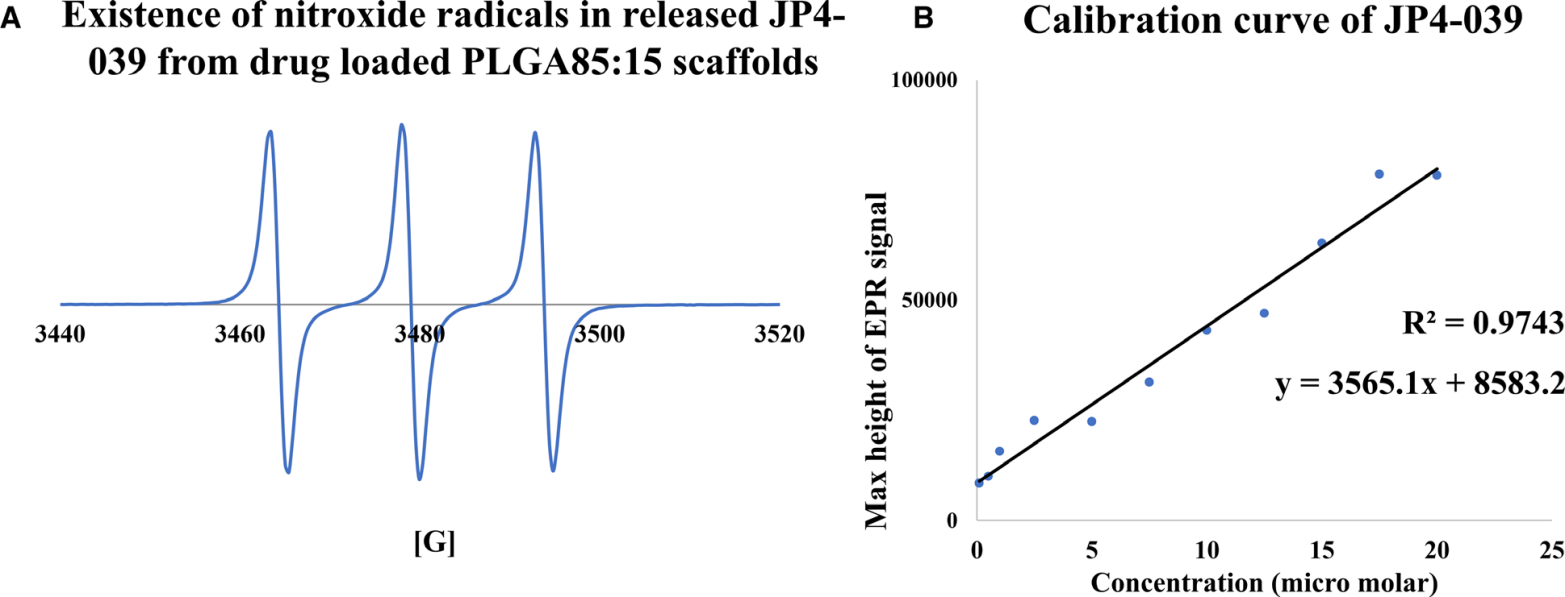
Electron paramagnetic resonance (EPR) and calibration curve of JP4‐039‐loaded PLGA85 : 15 nanofibrous scaffolds. (A) EPR spectra of JP4‐039‐loaded PLGA 85 : 15 scaffold dissolved in TFE showing nitroxide triplet peaks demonstrating the perseverance of active moiety of the drug after exposure to high electrostatic field in the fabrication technique. (B) Calibration curve of JP4‐039 using EPR spectroscopy to quantify the amount of the release drug from the scaffold by demonstrating a linear trendline correlating EPR spectra to the JP4‐30 concentration and the equation best describing it.

**Fig. 5:**
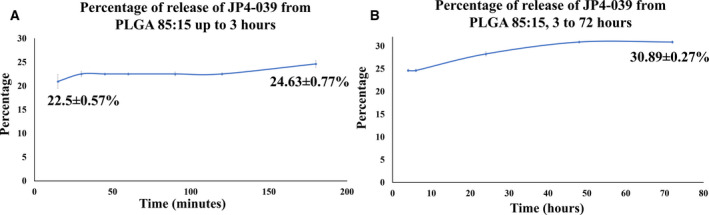
Release pattern of JP4‐039 from the nanofibrous scaffold. Percentage of cumulative release of JP4‐039 from drug‐loaded scaffold (A) up to 3 h (B) and up to 72 h showing an initial burst release of 22.5 ± 0.57% in initial 30 min following a more sustained release of 24.63 ± 0.77% in 3 h and 30.89 ± 0.27% in 72 h.

**Fig. 6:**
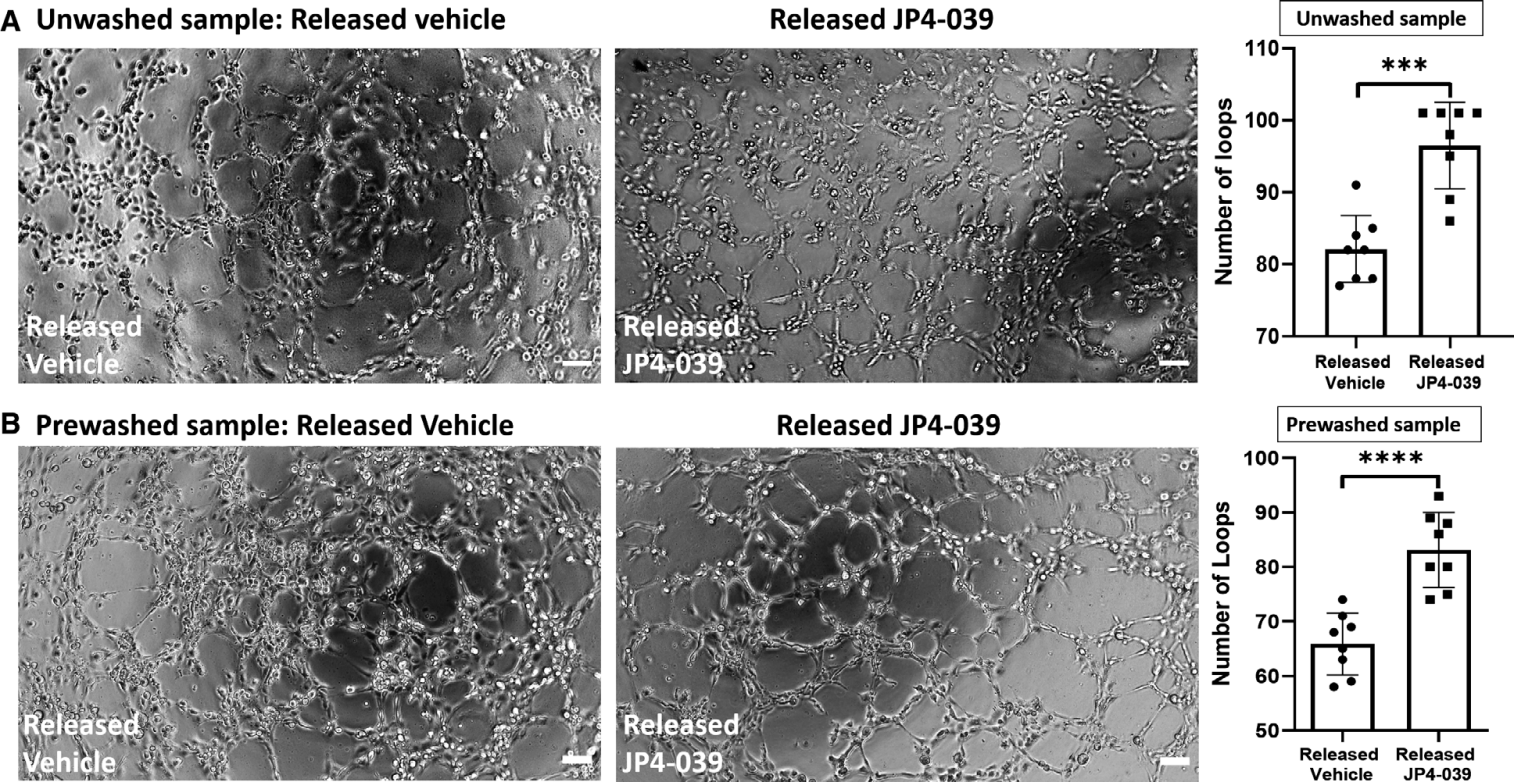
JP4‐039 released from scaffold increases tube formation in HCAEC. Representative images and graphical results show (A) the number of complete loops formed by HCAEC 3 h after incubation with JP4‐039 that was released from the nanofibrous scaffolds over a period of 24 h (unwashed sample), and (B) HCAEC 3 h after incubation with JP4‐039 that was released from a prewashed (for 20 min to get rid of the initial burst release containing high‐concentration JP4‐039) nanofibrous scaffold (prewashed sample). Two‐tailed Students' *t*‐test was carried out to determine statistical significance. *n* = 8; *P* = 0.001; results are expressed as mean ± standard error of the mean. Scale bar represents 100 µm.

Data were analyzed using two‐tailed Student's *t*‐test considering a *P‐*value inferior to 0.05 as significant. Results are expressed as mean ± standard error of the mean. Figures and graphs were prepared using Microsoft excel (Figs 2, 3, 4, 5) and graphpad prism 8 (GraphPad Software, San Diego, CA, USA) (Figs 1 and 6).

## Results

### JP4‐039 reduces mitochondrial ROS in HCAEC

In order to confirm mitochondrial specificity of the antioxidant activity of JP4‐039, HCAECs were incubated with or without 5 μm of JP4‐039 for 30 min and subject to a mitochondrial ROS measurement assay using the MitoSOX Red reagent as described in the manufacturer's protocol (ThermoFisher Scientific). JP4‐039 reduced mitochondrial ROS levels by 45 ± 14% compared to vehicle control (Fig. 1).

### Morphological characterization of electrospun scaffolds

We then analyzed the morphology and the diameter distribution of the electrospun polymeric fibers with and without enclosed JP4‐039 (Fig. 2A–D and Table 1). Scanning electron microscopy pictures of the electrospun polymeric fibers demonstrated that, upon optimizing the process and solution parameters, continuous bead‐free fibers were obtained from the polymeric solutions with or without enclosed JP4‐039. Table 1 shows the electrospinning conditions as well as the average fiber diameter of each scaffold obtained using imagej software. PLGA 85 : 15 polymer (with 85% PLA and 15% PGA) was spun into continuous bead‐free fibers at 2% wt concentration using a flow rate of 0.2 mL·h^−1^, a voltage of 15 kV and with a capillary to collector distance of 17 cm (Table 1). The fiber diameter of PLGA 85 : 15 without the enlaced drug showed a positive skewed distribution with an average value of 408.19 ± 3.71 nm (Figs 2A,C). When 2% JP4‐039 was incorporated in the solution, the fiber diameter reduced to 272.67 ± 2.45 nm and the distribution became normal comparing to blank PLGA 85 : 15 fibers (Fig. 2B,D). The average diameter of the fibers decreased after loading of the drug, which can be due to changes in the properties of the electrospinning solution, such as the conductivity.

**Table 1:** Summary of electrospinning parameters used for fabrication of PLGA 85 : 15 scaffolds with and without JP4‐039.

Polymer–drug	Concentration (wt/wt)	Flow rate (mL·h^−1^)	Distance (cm)	Voltage (kV)	Average diameter (nm)
PLGA 85 : 15	2%	0.2	17	15	408.1959 ± 3.71
PLGA 85 : 15/JP4‐039	2% Polymer 2% Drug	0.2	17	15	272.6631 ± 2.4573

### Chemical characterization of the electrospun scaffolds

The FTIR spectra of the polymer PLGA, the drug JP4‐039, and the drug‐loaded polymeric scaffolds demonstrated preservation of the chemical structure of the drug and the polymer after electrospinning process (Fig. 3). In the JP4‐039 spectra, the peaks at 3300 cm^−1^ correspond to the N–H stretching for secondary amines, which can also be seen in the JP4‐039‐loaded PLGA85 : 15 fibers (Fig. 3B). The peaks around 3000 cm^−1^ correspond to C–H stretching in alkenes and the ones at 2800 cm^−1^ for alkanes, the ones at 1680 cm^−1^ and 1650 cm^−1^ correspond to C=O stretching and N–H bonds, respectively, and the peaks at 1400–1500 cm^−1^ correspond to the N–O of the nitro compound of the drug which are all observed in the JP4‐039 spectra and JP4‐039‐loaded PLGA 85 : 15 fibers as well (Fig. 3A). For PLGA 85 : 15 fibers with and without the drug, the peaks at 2800–3000 cm^−1^ are related to the C–H stretching of the aliphatic part and OH of the carboxylic part of the polymer (Fig. 3B), and the peaks at 1700–1800 cm^−1^ correspond to C=O in carboxylic acid, and the peaks at 1450 cm^−1^ are for CH bending of methyl groups in the structure (Fig. 3A). Therefore, the FTIR spectra confirmed preservation of the chemical structure of JP4‐039 and PLGA 85 : 15 in the JP4‐039‐loaded PLGA 85 : 15 fibers postexposure to the electrostatic field during electrospinning method.

### Preservation of active moiety of the drug

The EPR spectra of JP4‐039‐loaded scaffold, dissolved in TFE were examined for the presence of nitroxide radicals in the electrospun scaffolds (Fig. 4A). The triplet peaks of the nitroxide radicals of JP4‐039 from the electrospun scaffold showed the presence of the radical scavenging active moiety in the fabricated patches (Fig. 4A). These peaks were identical to the original drug prior to loading onto the electrospun scaffolds, suggesting that the functional moiety of the drug was preserved in the PLGA nanofibrous scaffolds.

### Drug release from the electrospun scaffolds

The calibration curve of JP4‐039 was determined using EPR spectroscopy (Fig. 4B). A linear trendline could be plotted for the data and the equation correlating EPR spectra to JP4‐039 concentration was obtained while having *R* ^2^ value of 0.97. Using the equation derived from the calibration curve, the release of JP4‐039 from the PLGA 85 : 15 electrospun scaffold was analyzed for up to 72 h. Figure 5A,B shows the release pattern of JP4‐039 from the polymeric scaffolds up to 3 and 72 h, respectively. It was observed that released JP4‐039 had an initial burst release in the first 30 min following a more sustained release in the later hours. 22.5 ± 0.57% of the drug was released in the first 30 min; then till 3 h, it was at 24.63 ± 0.77%. Following this initial 3.5 h, the drug release increased very slowly over time. After 72 h, the cumulative drug release was 30.89 ± 0.27%. Together, these data suggest that JP4‐039 had an initial burst release from the PLGA 85 : 15 fibrous scaffold, followed by a sustained release over prolonged period of time.

### JP4‐039 released from the nanofibrous scaffold improved tube formation and wound healing in HCAEC *in vitro*

*In vitro* tube formation assay is a measure for EC proliferation and migration. When incubated in the presence of 5 μm JP4‐039 that was released from the scaffolds for over 24 h (‘unwashed sample’), HCAEC demonstrated 18 ± 3% increase in tube formation as compared to the vehicle‐treated HCAEC (released vehicle) (Fig. 6A). Control wells containing released vehicle had an average of 82 ± 1.62 tubes *versus* 96 ± 2.16 tubes in the wells treated with released JP4‐039 (Fig. 6A). In parallel experiments, another set of JP4‐039‐loaded scaffolds were subject to drug release where JP4‐039 that was released during initial burst release for 20 mins was discarded and the scaffolds were briefly washed, followed by collection of released JP4‐039 for over next 24 h (from > 20 min to 24 h) (‘prewashed samples’). In the presence of 5 µm JP4‐039 from prewashed samples (that lacks the initial burst release), HCAEC demonstrated 25.8% increase in tube formation (Fig 6B). Control wells containing released vehicle had an average of 66 ± 2.0 tubes *versus* 83 ± 2.4 tubes in the well treated with released JP4‐039 (Fig 6B). These findings suggest that JP4‐039 released from the nanofibrous scaffolds during the initial burst release as well as JP4‐039 released over a prolonged period of time can improve tube formation (i.e., proliferation and migration) in HCAEC significantly. In order to measure migration of EC, a wound healing, also known as scratch assay, was performed using HCAEC treated with 5 μm released JP4‐039 or released vehicle as control (Fig. S1). Released JP4‐039 improved healing (closure of the gap of the ‘scratch’) by 48 ± 8% and 22 ± 5%, after 3 and 6 h of induction of the scratch, respectively, as compared to cells treated with the released vehicle from the scaffold. Taken together, the findings suggest that the released JP4‐039 could enhance proliferation and migration in HCAEC.

## Discussion

In the current study, we have demonstrated that FDA‐approved biocompatible polymer PLGA 85 : 15 can be successfully used to fabricate a nanofibrous scaffold containing mitochondrial antioxidant JP4‐039 using electrospinning technique. The drug‐loaded scaffolds had continuous and bead‐free morphology. The chemical structure and functionality of the encapsulated JP4‐039 were conserved during the electrospinning procedure as shown by the FTIR analysis. The active moiety of the encapsulated drug, a nitroxide radical, was conserved in the structure of the released drug from the scaffold as shown by the EPR spectroscopy. Together, these findings suggest that the released drug, JP4‐039, from the scaffold possesses mitochondrial radical scavenging properties. The released drug also improved tube formation and wound healing in HCAEC *in vitro* suggesting that the reduction in mitochondrial ROS by JP4‐039 released from scaffolds induced EC proliferation and migration.

Electrospinning provides flexibility of delivery for drugs. In the current study, JP4‐039 could achieve up to 20.94 ± 1.49% release in the medium during the first few minutes (~ 20 min for our study) and then increased up to 30.89 ± 0.27% (cumulative) until 72 h followed by a more sustained release for a longer period of time. The burst release in the initial minutes is due to the diffusion of the drug into the surrounding media and the gradual 10% release in 72 h following a more sustained release is due to the degradation of the polymeric scaffold as the main mechanism of release. Therefore, the 70% remainder of the drug would get released from the scaffold over a longer period of time based on the degradation behavior of the polymeric scaffold. It has been reported that PLGA 85 : 15 degrades gradually within 30 weeks. Therefore, JP4‐039‐loaded scaffold would likely be able to provide an initial burst release followed by a sustained release of JP4‐039 for a prolonged period of time.

Electrospinning is a tunable process. The fiber diameter obtained for the current study with loaded JP4‐039 is the result of optimizing various parameters. According to the literature, changes in the electrospinning parameters lead to an alteration in the final fiber morphology. An increase in the flow rate of the solution results in the formation of fibers with larger diameters because, with the same applied voltage, the fiber jet gets stretched less thereby resulting in thicker fibers. In the same way, decreasing the applied voltage when flow rate is kept constant forms larger fibers, since fibers are getting less stretched, and therefore less thinning happens during their flow to the grounded collector. It is expected to get reduced release of the drug from fibers with larger diameters and the same porosity, due to a decrease in the final fiber surface area, although often a combination of morphological changes in the fibers such as diameter and degree of porosity of the fiber would determine the final release pattern [38, 39, 40, 41]. Therefore, it is possible to load desired concentration of the drug and achieve an optimal release pattern by modulating electrospinning and morphological parameters, without requiring to change the total quantity of the polymer used and/or dimension of the scaffold. For instance, as long as the concentration of the drug is below the saturation point in the solution, the initial drug to polymer ratio can be tailored in order to encapsulate a larger amount of the drug inside the fibers and consequently obtain a higher concentration in the released medium. For the applications requiring a drug to be delivered to the tissue promptly, utilization of a polymer with appropriate hydrophilic properties can be used to obtain a faster release behavior [42]. On the contrary, a polymer with a more hydrophobic nature can be used for providing a more sustained release. In summary, electrospinning is a system which is capable of being tuned to provide the required release/delivery pattern of a drug. This is the most exciting advantage of using electrospun polymeric scaffolds as implant‐based drug delivery system.

In previous work by others, JP4‐039 has been enclosed into microneedle assays (MNAs) and used as a radiomitigator for topical delivery in mice exposed to body irradiation, and compared to normal IV injections [43]. In the injection method, JP4‐039's concentration in blood increased and then rapidly decreased after 5 min and was stabilized after 10 min. In contrast, MNAs provided a slow increase in the blood concentration for the first 5 min and then stayed at the same level as IV injection [43]. JP4‐039 has also been injected in mice at 1 mg·kg^−1^ dosage and incubated with 32D cl 3 murine hematopoietic progenitor cells at 1 μm prior to exposure to irradiation, respectively, and showed a significant increase in the survival rate [44]. In another study, the effect of JP4‐039 has been evaluated in a radiation‐induced skin damage model by applying 50 μL of JP4‐039 with or without the lipid carrier at 30 min, 24 h, and 48 h after irradiation [23]. In the present study, biocompatible, biodegradable JP4‐039‐loaded nanofibrous patches offer the advantage of a high concentration of the drug within the first 3 h followed by a sustained release over the next 72 h. These biocompatible nanofibrous scaffolds capable of releasing an uploaded drug over a prolonged period of time would replace the need for reapplication or multiple injections of the drug. Future *in vivo* studies will be required to examine whether the implanted nanofibrous patches will deliver JP4‐039 to the impacted area in a sustained and controlled release pattern. Removal of a patch will not be required since FDA‐approved biodegradable polymers (PLGA) have been used for fabrication of the scaffold as the drug reservoir and delivery. Furthermore, as mentioned earlier electrospun nanofibrous scaffolds are tunable, that is, the fibrous structure can easily be manipulated and the required release behavior can be altered depending on the desired treatment [42, 45, 46, 47]. At a functional level, JP4‐039 released from the nanofibrous scaffolds over a period of 24 h improved tube formation (a function of migration and proliferation) (Fig. 6A) and ‘wound healing’ (a measure of migration) in HCAEC (Fig. S1), suggesting the applicability of the fabricated patches in reducing oxidative stress and improving functionality in HCAEC. Our data also confirmed a query whether exclusion of the initial 20 min burst release from 24 h ‘prewashed samples’ of released JP4‐039 would still be functionally active (Fig. 6B). As we learn more about the molecular mechanisms of the complex nature of mitochondrial ROS in modulating EC metabolism and thus recovery in myocardial ischemia, we will need to devise a more precise and temporal modulation of antioxidant release in *in vivo* setting in future studies. Electrospinning provides this flexibility either through polymer–drug process parameter combination [29, 45, 48, 49, 50], or through controlled release using external actuation. [25, 31, 51]. Needless to mention, PLGA being FDA‐approved and biocompatible, has already marked safe to be used in *in vivo* assays and clinical settings for many applications which eliminate the concern of toxicity for our JP4‐039‐loaded scaffold [52, 53]. In a study where linezolid‐loaded PLGA scaffold was implanted in rats with bone and soft tissue infection, no sign of toxicity was observed [54]. The PLGA used in that study was a 1 × 1 square centimeter scaffold weighing 12 mg, which is much larger than the scaffolds used in our current study, and showed no signs of toxicity. For our future *in vivo* studies, based on the concentration requirement of JP4‐039, about 1–3 μg of the drug and 30 μg of polymer scaffold will be needed to obtain a sustained release in mouse models. Thus, we do not anticipate any toxicity to be observed in our *in vivo* models. Additionally, PLGA and PLGA‐based fibrous scaffolds, due to their biocompatibility, have also been used in other studies for *in vivo* evaluations [55].

In conclusion, the JP4‐039‐loaded continuous fibrous patches, made from electrospun FDA‐approved biodegradable polymers, are capable of preserving the mitochondrial‐targeting antioxidant activity of the drug and can act as a source to deliver the drug over a prolonged period of time. We reported earlier that reduction in mitochondrial ROS improved *EC* proliferation *in vitro* and aortic angiogenic sprouting *ex vivo* in a transgenic animal model with oxidative stress in vascular endothelium [14]. In the current study, an enhancement in EC proliferation and migration as shown by increased tube formation and wound healing *in vitro* by the mitochondria‐specific antioxidant drug JP4‐039 released from the nanofibrous scaffolds also supported a positive role for reduction in mitochondrial ROS in HCAEC. Therefore, the JP4‐039‐loaded PLGA 85 : 15 fibrous scaffolds appear to be good candidates for conditions that require prolonged reduction in mitochondrial ROS. In future studies, the activity and efficacy of the drug‐encapsulated biodegradable implants will be evaluated *in vivo* using animal models of oxidative stress and ischemia.

## Conflict of interest

PW is a named inventor on patents held by the University of Pittsburgh covering JP4‐039 composition and use.

## Author contributions

YH performed experiments, acquired and analyzed data, and wrote the paper; RBT performed experiments, analyzed data, and cowrote the paper; CK performed data analysis and graphical abstract and edited part of the paper; PW supplied JP4‐039, helped troubleshoot, and edited paper; SB helped with experiments, performed data analysis, and edited the paper; MRA conceptualized the study, supervised experiments, interpreted data, edited, finalized, and submitted the paper.

## Supporting information


**Fig. S1.** JP4‐039 released from scaffold increases wound healing in HCAEC. Representative images and graphical results show the increase in area difference (decrease in wound area) at zero (left panel), three (middle) and six hours (right) after the scratch. Upper panels show HCAEC incubated with Vehicle released from the unloaded scaffolds, and lower panels show HCAEC incubated with JP4‐039 released from the drug‐loaded scaffolds over a period of 24 hours. Bar graphs show statistical analysis of ‘wound healing’ (scratch closure) or gap closure at 3 hours (left) and 6 hours (right) after the induction of the assays. Two‐tailed Students' t‐test was carried out to determine statistical significance. n=8; *p=0.0014 (0‐3 hrs)*, *p=0.0076 (0‐6 hrs)*. Results are expressed as mean ± standard error of the mean. Scale bar represents 100µm.

## Data Availability

Data will be available from the corresponding author upon reasonable request.
