# Supplementary figures and images for: Delivery of a mitochondria‐targeted antioxidant from biocompatible, polymeric nanofibrous scaffolds
Source: FEBS Open Bio. 2020 Dec 8;11(1):35–47. doi: 10.1002/2211-5463.13032 (PMC7780095; doi:10.1002/2211-5463.13032)

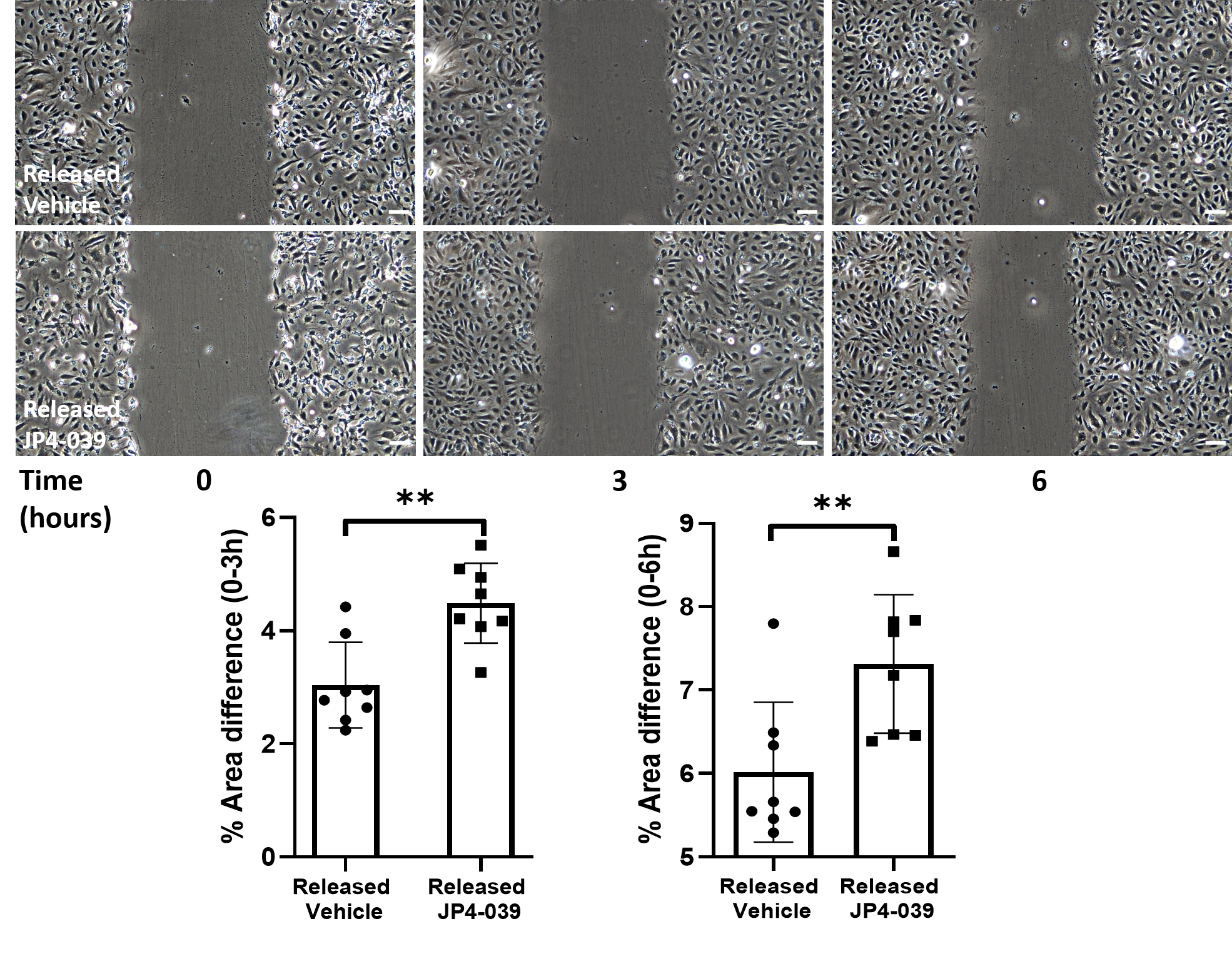

Supplement: Supplementary file 1 — Fig. S1. JP4‐039 released from scaffold increases wound healing in HCAEC. Representative images and graphical results show the increase in area difference (decrease in wound area) at zero (left panel), three (middle) and six hours (right) after the scratch. Upper panels show HCAEC incubated with Vehicle released from the unloaded scaffolds, and lower panels show HCAEC incubated with JP4‐039 released from the drug‐loaded scaffolds over a period of 24 hours. Bar graphs show statistical analysis of ‘wound healing’ (scratch closure) or gap closure at 3 hours (left) and 6 hours (right) after the induction of the assays. Two‐tailed Students' t‐test was carried out to determine statistical significance. n=8; p=0.0014 (0‐3 hrs), p=0.0076 (0‐6 hrs). Results are expressed as mean ± standard error of the mean. Scale bar represents 100µm. [file FEB4-11-35-s001.tif]
